# Medical concepts related to individual risk are better explained with "plausibility" rather than "probability"

**DOI:** 10.1186/1471-2261-5-31

**Published:** 2005-09-27

**Authors:** Enzo Grossi

**Affiliations:** 1 Medical Department, Bracco SpA Milan, Italy

## Abstract

**Background:**

The concept of risk has pervaded medical literature in the last decades and has become a familiar topic, and the concept of probability, linked to binary logic approach, is commonly applied in epidemiology and clinical medicine. The application of probability theory to groups of individuals is quite straightforward but can pose communication challenges at individual level. Few articles by the way have tried to focus the concept of "risk" at the individual subject level rather than at population level.

**Discussion:**

The author has reviewed the conceptual framework which has led to the use of probability theory in the medical field in a time when the principal causes of death were represented by acute disease often of infective origin.

In the present scenario, in which chronic degenerative disease dominate and there are smooth transitions between health and disease the use of fuzzy logic rather than binary logic would be more appropriate. The use of fuzzy logic in which more than two possible truth-value assignments are allowed overcomes the trap of probability theory when dealing with uncertain outcomes, thereby making the meaning of a certain prognostic statement easier to understand by the patient.

**Summary:**

At individual subject level the recourse to the term *plausibility*, related to fuzzy logic, would help the physician to communicate to the patient more efficiently in comparison with the term *probability*, related to binary logic. This would represent an evident advantage for the transfer of medical evidences to individual subjects.

## Background

The concept of risk has pervaded medical literature in the last decades and has become a familiar topic. Few articles by the way have tried to focus the concept of "risk" at the individual subject level rather than at population level [1]. The concept of medical risk for the single individual from a mathematical point of view opens an interesting philosophical debate on the appropriate use of the term *probability*.

As I will try to explain, the recourse to the term *plausibility*, related to fuzzy logic, would help the physician to communicate to the patient more efficiently in comparison with the term *probability*, related to binary logic.

The dictionaries tell us that risk is "the possibility of loss or injury". This definition is familiar to most of us when we think of the possibility of being involved in a crash when driving a car or when flying in a plane.

In medicine like in many other contexts the assessment of a particular risk related to the occurrence of a harmful event is generally performed by means of probability theory.

Medical science has borrowed this approach from other disciplines, like astronomy, fine arts, gambling, and insurance, which contributed most to the development of the mathematics of probability already back in the 17^th ^century. Still today, for well defined random events, the probability distribution can easily be determined employing methods originally developed by Blaise Pascal [2].

One should not be surprised by this fact, since the development of the modern science of medicine took place when the prevalent causes of death were infectious diseases which typically follow an epidemic behavior. It is evident that when dealing with pathogen transmission a certain degree of randomness clearly exists, and this justifies the recourse to probability theory.

Over the last fifty years, at least in western countries, the overall health scenario has dramatically changed, and cardiovascular diseases, cancer, and degenerative diseases have gradually dominated the health scenario, overtaking infectious diseases as the principal cause of death.

These chronic conditions behave as complex systems dominated by gradual onset over time and multivariate causes changing over time. More specifically, events occurring in association with these diseases definitively have an explanation, and are seldom, almost never, truly random. The problem, if any, lies in the complexity of this explanation which sometimes exceeds our present understanding, forcing us to use sophisticated modeling.

## Discussion

### Plausibility vs probability

The use of probability theory to assess the risk of undergoing a "cardio-vascular event" for example would mean that the event takes place as a "all or nothing" phenomenon, while it is generally not.

An "all or nothing" phenomenon implies that the condition of the subject exposed to the risk of the event does not change in relation to the actual occurrence of the event. For example, if the event consists in being hit by a tile falling from a roof whilst walking along a street, we wouldn't expect particular transition phases preceding the unfortunate event, at least at the level of the victim of the event. In clinical settings on the contrary, very often, even if the event takes place suddenly, resembling a falling tile, it can be considered as the natural final outcome of an unstable and evolving condition which predisposes by its nature the subject to the event. In order to better explain this concept we can consider the case of a cerebro-vascular event in relation to the presence of a carotid stenosis. We know that local lesion parameters (morphology, degree of stenosis), hemodynamic factors (collateral compensation) and systemic factors (clinical symptoms, accompanying diseases, risk factor control) have been taken into account to develop a model able to determine the likelihood of the occurrence of an event. To simplify the reasoning, we can assume that it is just the degree of stenosis which actually influences and ultimately determines the development of the event. For example, when the degree of stenosis reaches and exceeds a definite level, suppose > 90% of the vessel lumen, then the occurrence of the event becomes almost unavoidable. Following this reasoning, a patient with 70% stenosis, while being perfectly asymptomatic has a probability of having an event, within a certain time-frame, of 80%, while for a patient with 50% stenosis the same value could decrease to 30%. The patient in this example would make a transition along different degrees of event "plausibility", by evolving with their asymptomatic carotid disease, while the subject walking along the street would remain continuously in a "all or nothing" situation. In the latter case one would use the frequency of being hit on the head by a falling tile in a general population to describe the risk to which this subject is exposed, for example 1: 100.000, while in the other case a better definition would be the membership degree of the patient to the typical condition predisposing to the event, a concept which is described more clearly using fuzzy logic, a special multivalent logic, rather than binary logic.

As it is known, standard logic applies only to concepts that are completely true (having degree of truth 1.0) or completely false (having degree of truth 0.0), deriving directly from Aristotelian law of the "excluded middle".

Traditionally, logical calculi are bivalent that is, there are only two possible truth values for any proposition, true and false (which generally correspond to our intuitive notions of truth and falsity). But bivalence is only one possible range of truth values that may be assigned, and other logical systems have been developed with variations on bivalence, or with more than two possible truth-value assignments. In the classical bivalence scheme, true and false are determinate values: a proposition is either true or false (exclusively), and if the proposition does not have one of those values, by definition it must have the other. This is the justification for the Law of excluded middle: P`/¬P (i.e., either the proposition or its negation holds).

Fuzzy logic is a generalization of standard logic, in which a concept can possess a degree of truth anywhere between 0.0 and 1.0.

### Fuzzy logic vs probability

Fuzzy logic was originally intended to be used for reasoning about inherently vague concepts, such as 'height.' or "age" [3]. For example, we might say that 'President Berlusconi is tall,' with a degree of truth of 0.6.

At this stage it is important to point out the distinction between fuzzy systems and probability. Both operate over the same numeric range, and at first glance both have similar values: 0.0 representing False (or non- membership), and 1.0 representing True (or membership). However, there is a distinction to be made between the two statements: The probabilistic approach yields the natural-language statement, "There is an 80% chance that Nelson Mandela is old," while the fuzzy terminology corresponds to "Nelson Mandela's degree of membership within the set of old people is 0.80." The semantic difference is significant: the first view supposes that Nelson Mandela is or is not old; it is just that we only have an 80% chance of knowing which set he is in. By contrast, fuzzy terminology supposes that Mandela is "more or less" old, and in the specific example the "fuzzy degree" of membership corresponds to the value of 0.80.

It is important to reiterate that at mathematical level, fuzzy values can be easily misunderstood to be probabilities, and that one can believe that fuzzy logic is a fancy way to handle probabilities.

A fundamental difference is that while the sum of probabilities of two disjoint sets must always be equal to one (requirement of additivity), fuzzy measures can be either super or subadditive.

In other words sets that are fuzzy, or multivalent, break the Aristotelian law of the excluded middle and can belong only partially to a fuzzy set(sub-additive) or also belong to more than one set (super-additive). As clearly stated by Kosko & Isaka, fuzzy degrees are not the same as probability percentages. Probability measures whether something will occur or not. Fuzziness measures the degree to which some condition exists or something occurs [4].

For the readers interested to deepen the topic of the differences between probability and fuzzy theory the essay written by George Klir represents an essential reference [5].

### Fuzzy logic in science and medicine

Although traditional statistics systems based on binary logic have been used successfully as diagnostic decision aids in different fields of medicine, it is now more and more evident that their obliged recourse to probability theory to represent uncertainty in the medical context may be inappropriate in many circumstances and partly responsible for their limitations in certain applications.

In recent years interesting proposals for the application of fuzzy logic in medical science have appeared in the literature, and in this regards a special mention has to be made to the contributions coming from the group of Helgason, aiming for example to better individualize diagnostic process[6] or prescribing and dosing of particular medications at the bedside[7,8].

An interesting concept among others emerging from these papers is that fuzzy measures are better able to capture variables interactions in the individual subjects in comparison with standard statistical measures [9]. Other authors have also proposed the use of fuzzy logic to concepts to population biology with emphasis in epidemiological problems like causal studies, epidemic models and designing of vaccination strategies [10], showing that the applications of fuzzy sets in epidemiology is a very promising area of research.

But what about prognosis? Could we use fuzzy logic in the presence of occurrence facts?

My tentative answer would be "yes".

In the cardiovascular field of medicine this could make a substantial difference. In the case of a probabilistic approach we would have to inform the patient that given their present clinical condition,(i.e. a carotid stenosis of 70% showed at B mode ultrasound), he has an 80% probability of undergoing an event within a certain time-frame.

In other words, the patient will be told that since 80% of patients previously diagnosed with a similar clinical condition underwent an event within a given timeframe, this population has an average risk of 80%.

At this point the patient may well ask the physician whether at the moment he belongs to the 80% or to the 20% subgroup, putting the physician in a difficult position.

### Summary

The physician would be paradoxically more precise with fuzzy terminology: he could explain to the patient that, given his present clinical condition demonstrated by a B mode ultrasound, he has reached 80% of the course between the previous safe condition and a future unavoidable event, as one would explain to a man who, without noticing it, is progressing step by step from a safe point to the edge of a cliff.

The use of fuzzy logic could allow to escape the probability theory trap in order to deal with a certain degree of uncertainty, thereby making the meaning of a certain prognostic statement easier to understand by the patient.

This would represent an evident advantage for the transfer of knowledge of diseases to individual subjects.

## Competing interests

The author(s) declare that they have no competing interests.

## Pre-publication history

The pre-publication history for this paper can be accessed here:


